# Supplementary material for: Functional Genes and Transcripts Indicate the Existent and Active Microbial Mercury-Methylating Community in Mangrove Intertidal Sediments of an Urbanized Bay
Source: Microorganisms. 2024 Jun 20;12(6):1245. doi: 10.3390/microorganisms12061245 (PMC11205478; doi:10.3390/microorganisms12061245)
Supplement: Supplementary file 1 [file microorganisms-12-01245-s001.zip › microorganisms-3052961-supplementary.pdf]

Supplementary information

Table S1. Physicochemical parameters of the sampling sites in the intertidal sediments of Shenzhen Bay

**Parameter**	**Site1**	**Site2**	**Site3**
pH	6.67	6.72	6.83
Eh (mV)	-112	-107	-121
TOC (mg g^{-1})	6.98	7.13	7.4
TN (mg g^{-1})	0.68	0.75	0.65

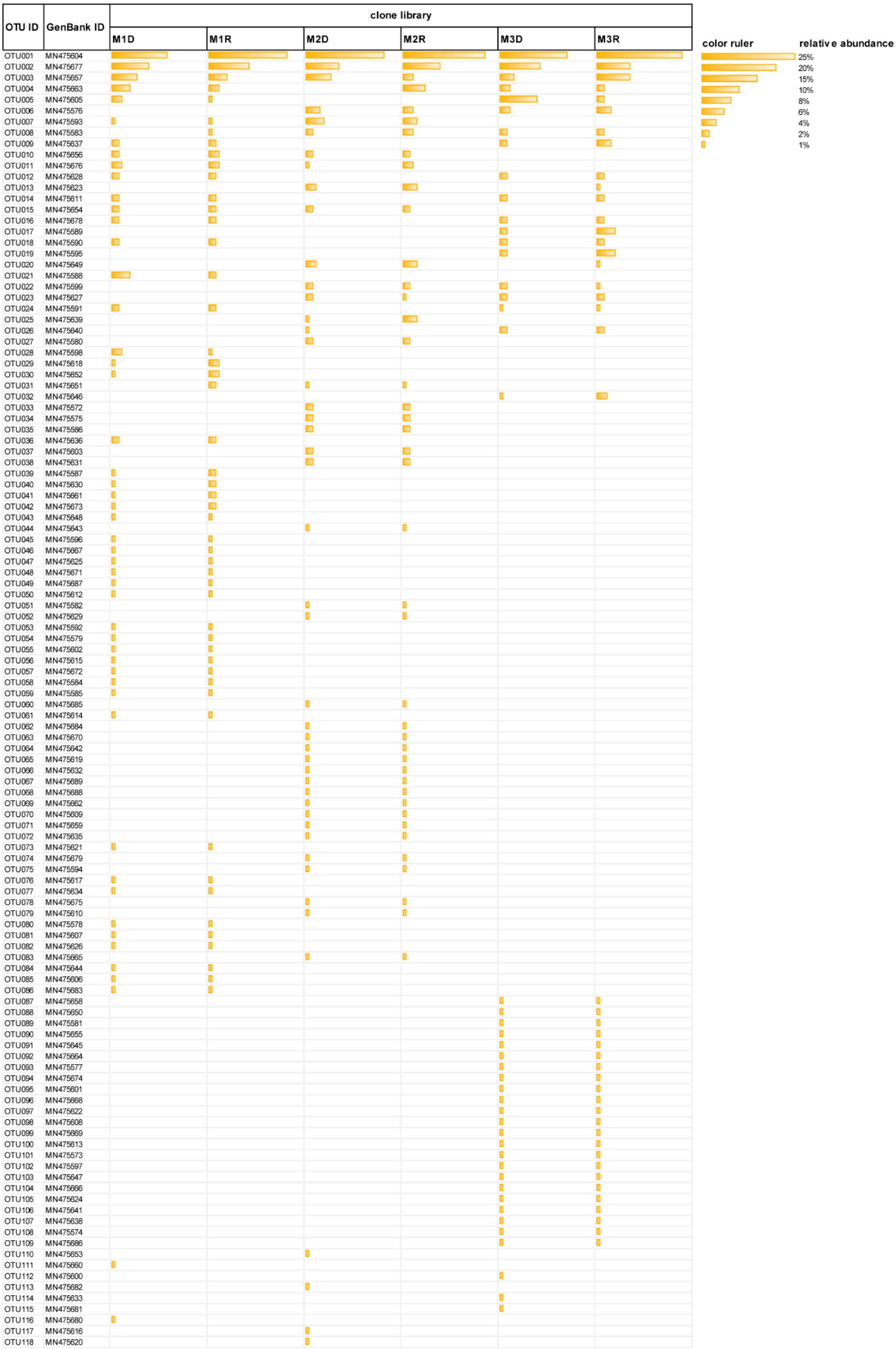

Figure S1. Relative abundance of each *hgcA* phylotype in corresponding clone library. M1D, M2D, M3D represent the DNA level clone libraries from sampling site1, site2, site3, respectively; M1R, M2R, M3R represent the RNA level clone libraries from sampling site1, site2, site3, respectively.
